# Discovering weaker genetic associations guided by known associations

**DOI:** 10.1186/s12920-020-0667-4

**Published:** 2020-02-24

**Authors:** Haohan Wang, Michael M. Vanyukov, Eric P. Xing, Wei Wu

**Affiliations:** 1 Language Technologies Institute, School of Computer Science, Carnegie Mellon University, Pittsburgh, PA USA; 2 Department of Pharmaceutical Sciences, Departments of Psychiatry, and Human Genetics, University of Pittsburgh, Pittsburgh, PA USA; 3 Machine Learning Department, School of Computer Science, Carnegie Mellon University, Pittsburgh, PA USA; 4 Computational Biology Department, School of Computer Science, Carnegie Mellon University, Pittsburgh, PA USA

**Keywords:** Weak association, Linear mixed model, GWAS

## Abstract

**Background:**

The current understanding of the genetic basis of complex human diseases is that they are caused and affected by many common and rare genetic variants. A considerable number of the disease-associated variants have been identified by Genome Wide Association Studies, however, they can explain only a small proportion of heritability. One of the possible reasons for the missing heritability is that many undiscovered disease-causing variants are weakly associated with the disease. This can pose serious challenges to many statistical methods, which seems to be only capable of identifying disease-associated variants with relatively stronger coefficients.

**Results:**

In order to help identify weaker variants, we propose a novel statistical method, Constrained Sparse multi-locus Linear Mixed Model (CS-LMM) that aims to uncover genetic variants of weaker associations by incorporating known associations as a prior knowledge in the model. Moreover, CS-LMM accounts for polygenic effects as well as corrects for complex relatednesses. Our simulation experiments show that CS-LMM outperforms other competing existing methods in various settings when the combinations of MAFs and coefficients reflect different scenarios in complex human diseases.

**Conclusions:**

We also apply our method to the GWAS data of alcoholism and Alzheimer’s disease and exploratively discover several SNPs. Many of these discoveries are supported through literature survey. Furthermore, our association results strengthen the belief in genetic links between alcoholism and Alzheimer’s disease.

## Background

Genome Wide Association Studies (GWAS) have allowed people to address one of the most fundamental tasks in genetic research, which is to uncover associations between genetic variants and complex traits. Many efforts have been made which employ traditional statistical testing methods such as the Wald test to test the association of each individual SNP with a certain human disease, yet there are still a large amount of missing heritability to be discovered [1], which is due to the relatively low statistical power of these methods. In order to increase the power of the association mapping, many statistical approaches have been proposed.

For example, linear regression and the Lasso variants have been introduced to account for polygenic effects commonly seen in complex human diseases [2, 3]. Following the success of Lasso methods, the Adaptive Lasso with the oracle property under some regularity conditions [4], and the Precision Lasso that works with correlated and linearly dependent variables [3] were proposed.

However, a natural limitation of the Lasso-based approaches is that they do not account for confounding effects raised by population structure and other complex relatedness in the GWAS data. In order to correct such effects, linear mixed models (LMMs) have been developed and received much attention in the recent years [5, 6]. Recently, Segural *et al* introduced a multi-locus LMM that utilizes step-wise selection to model polygenetic effects [7]. Further Liu *et al* extended the multi-locus LMM by dividing the model into fixed effect model and random effect model and use them iteratively [8]. On an alternative approach, recent studies also proposed a multi-locus extension to the standard LMM to account for polygenic effects with the introduction of priors on coefficients [9, 10].

Despite the success of the aforementioned methods achieved, these methods are not effective in identifying genetic variants with weaker coefficients. Considering the current notion that many complex human diseases are likely to be caused and affected by many–rather than a few–genetic variants with small coefficients on a certain disease [11] and yet only a limited number of significant disease-associated variants have been identified from GWAS, we conjecture that the variants with small coefficients are difficult to identify given the presence of the variants with much larger coefficients, and that they will become easier to detect when conditioning on frequently reported SNPs which usually have larger coefficients. Following this belief, we propose a novel statistical method, Constrained Sparse Multi-locus Linear Mixed Model (CS-LMM), [12, 13] to uncover novel genetic variants of smaller coefficients by: 1) incorporating those frequently reported or known variants as a prior knowledge to the model, 2) accounting for polygenic association with a multivariate sparse regularized regression, and 3) correcting for population structure and complex relatedness (including family structure and other cypticx relatedness).

The performance of the CS-LMM model is evaluated using extensive simulation experiments. We also apply our CS-LMM model to an alcoholism and an Alzheimer’s Disease GWAS data, with the prior knowledge of the reported SNPs associated with each disease. We identify a set of SNPs having weak associations with each disease. Most of our findings are consistent with previously published results.

## Methods

We formally introduce our model named Constrained Sparse Multi-locus Linear Mixed Model (CS-LMM) that aims to uncover genetic variants with weaker associations of a disease by incorporating variants of known associations as a prior knowledge.

### Model

Given frequently reported or known variants (will be called known variants later for simplicity) with relatively larger coefficients, our model CS-LMM aims to uncover novel variants of smaller coefficients. In order to achieve this, let **X** denote genotype data, **Z** denote population identification, **y** denote phenotype data (we first assume quantitative traits here, and discuss the case-control data or binary traits later), and let $\mathcal {K}$ denote the set of the variants that are known or frequently reported. The “coefficient” is mathematically defined as the coefficient of linear regression [14]. With these settings, we have our CS-LMM model formally presented as:

$$\begin{array}{*{20}l} & \mathbf{y} = \mathbf{X}\beta + \mathbf{Z}\mathbf{u} + \epsilon \\ & \mathbf{u} \sim N(0, \mathbf{I}\sigma_{u}) \\ & \epsilon \sim N(0, \mathbf{I}\sigma_{\epsilon}) \\ & \textnormal{subject to } \quad ||\beta||_{1} \leq c, \\ & |\beta_{i}|>0, \quad \forall i \in \mathcal{K}, \\ & |\beta_{j}|<|\beta_{i}|, \quad \forall i \in \mathcal{K}, j \notin \mathcal{K} \end{array} $$

where *β* is the fixed genetic effects; *u* denotes the random population effects; *ε* is natural noise. We also introduce a constraint term ||*β*||_1_≤*c* with the belief that only a subset of the SNPs are associated with the phenotype, where *c* is a constant.

### Algorithm

We proceed to introduce a three-phase algorithm to estimate the parameter *β*, *σ*_*u*_, and *σ*_*ε*_ in the CS-LMM model.

- **Step I. Fitting known variants of larger coefficients**: We first fit a linear regression model to determine the coefficients (magnitude of *β*_*i*_) for the known SNPs, by solving the following equation:
  1 $$\begin{array}{*{20}l} \hat{\beta_{i}} = \arg\min_{\beta_{i}} ||\mathbf{y}-\sum_{i}\mathbf{X}^{i}\beta_{i}||_{2}^{2}, \quad \forall i \in \mathcal{K}  \end{array} $$
- **Step II. Correcting for population stratification and complex relatedness**: Then, we consider to estimate *σ*_*u*_ and *σ*_*ε*_ for population stratification. Since **y**=**X***β*+**Zu**+*ε* (**u**∼*N*(0,*σ*_*u*_) and *ε*∼*N*(0,*σ*_*ε*_)) is equivalent to $\mathbf {y} \sim N(\mathbf {X}\beta, \mathbf {Z}\mathbf {Z}^{T}\sigma _{u}^{2}+I\sigma _{\epsilon }^{2})$, we can estimate the variance term with a maximum likelihood estimation of Gaussian distribution by maximizing the following:
  2 $$\begin{array}{*{20}l} l(\sigma_{u}, \sigma_{\epsilon} | \mathbf{y}', G) \propto N(\mathbf{y}' - \bar{\mathbf{y}'} | 0, \sigma_{u}^{2}\mathbf{Z}\mathbf{Z}^{T} + \sigma_{\epsilon}^{2}\mathbf{I})  \end{array} $$
  where $\bar {\mathbf {y}'}$ is the empirical mean of *y*^′^ that is calculated by
  3 $$\begin{array}{*{20}l} \mathbf{y}' = \mathbf{y} - \sum_{i} \mathbf{X}^{i}\hat{\beta_{i}}  \end{array} $$
  and **ZZ**^*T*^ is the genomic relationship matrix that is estimated as **ZZ**^*T*^=(**X**^*j*^)(**X**^*j*^)^*T*^, following the convention [15].
  We then solve Eq. 2 for *σ*_*u*_ and *σ*_*ε*_, where we can adopt the trick of introducing $\delta = \frac {\sigma _{\epsilon }^{2}}{\sigma _{u}^{2}}$ to replace $\sigma _{u}^{2}$ for more efficient optimization [16].
  Finally, we can correct the population stratification by rotating the original data:
  $$\begin{array}{*{20}l} \tilde{\mathbf{X}^{j}} &= (\textnormal{diag}(\mathbf{\Gamma})+\delta \mathbf{I})^{-\frac{1}{2}}\mathbf{V}^{T}\mathbf{X}^{j} \\ \tilde{\mathbf{y}'} &= (\textnormal{diag}(\mathbf{\Gamma})+\delta \mathbf{I})^{-\frac{1}{2}}\mathbf{V}^{T}\mathbf{y}' \end{array} $$
  where **ZZ**^*T*^=**UΓV**^*T*^ is the singular value decomposition.
- **Step III. Fitting variants with smaller coefficients**: Finally, we try to use the rest SNPs to explain the residual phenotypes, with solving the following:
  $$\begin{array}{*{20}l} \hat{\beta_{j}} =& \arg\min_{\beta_{j}} ||\tilde{\mathbf{y}'}-\sum_{j}\tilde{\mathbf{X}^{j}}\beta_{j}||_{2}^{2} \\ & \textnormal{subject to}\quad |\beta_{j}| < \min|\beta_{i}|, \quad \forall j \quad \forall i \end{array} $$
  To solve this problem efficiently, we relax this constrain to a Lasso constrain as follows:
  4 $$\begin{array}{*{20}l} \hat{\beta_{j}} =& \arg\min_{\beta_{j}} ||\tilde{\mathbf{y}'}-\sum_{j}\tilde{\mathbf{X}^{j}}\beta_{j}||_{2}^{2} + \sum_{j}\lambda||\beta_{j}||_{1}  \end{array} $$
  This new Lasso problem is solved via proximal gradient descent [17].
  **Stability Selection** In Step III, to achieve a stable variable selection, we follow the regime of stability selection [18]: we run the algorithm 100 times, each time with half of the data points sampled without replacement from the original data. The final selected variables are the ones that are chosen more than 75% of chances over 100 runs.

### Implementation

The implementation of CS-LMM is available as a python software. Without installation, one can run the software with a single command line. It takes the Plink binary data as input. An extra file containing the known association variants is recommended. If this extra file is not available, CS-LMM will first employ standard testing methods such as Wald test to select variants with the strongest signals. In order to identify a specific number (denoted as *K*) of SNPs associated with the disease, users can inquire the model with the number *K* or with a specific weight of the regularization term (*λ* in Eq. 4). If neither the number of SNPs nor the regularization weight is specified, the software will estimate the parameters using cross validation. The detailed instruction on how to use the software can be found in the Additional file 1. The implementation is available as a standalone software^1^. The computational complexity and scalability scales linearly with the number of samples and SNPs.

## Results

### Simulations

In order to evaluate the performance of CS-LMM, we compare it with several existing association methods regarding their ability to uncover weaker associations. In particular, we compare CS-LMM to the following methods:

- Standard Wald test with the standard FDR control using the Benjamini–Hochberg (BH) procedure [19]: the most popular test used in GWA studies;
- L1-regularized linear regression (i.e. the Lasso);
- Adaptive Lasso: an extension of Lasso that weighs the regularization term [4] (enabled by the method introduced in [20] for high-dimensional data);
- Precision Lasso: a novel improvement of Lasso that is more stable and consistent than Lasso [3];
- Linear mixed model: the most popular method of population stratification;
- Sparse linear mixed model (sparse LMM): a combination of sparse variable selection and population stratification [9, 21].
- Multi-locus linear mixed model (MLMM): an improvement of linear mixed model with step-wise selection to enable polygenetic modelling [7].
- Fixed and random model Circulating Probability Unification (FarmCPU): a novel extension of MLMM that iteratively uses fixed effect model and random effect model [8]

#### Data generation

We generate the simulation data comprehensively to reflect real world scenarios of genetic data with population structure under different minor allele frequencies (MAFs) and coefficients. We use the SimuPop [22] software to simulate the real world genomic data with population structure. We simulate *p* SNPs for *n* individuals, denoted as **X**, and let **X**^*j*^ denote the *j*^th^ SNP. These individuals are from *g* populations and each population has *f* subpopulation.

In our simulation experiments, the SNPs come from two sets with two different MAFs: 20*%* of these SNPs are from one set (denoted as Set *v*) which has an MAF as *m*_*v*_ while the rest of the 80*%* SNPs are from the other set (denoted as Set *u*) which has a MAF as *m*_*u*_. We assume there are *k* SNPs associated with the phenotype, of which, 20*%* are from set *v* and the rest are from set *u*.

**Fig. 1:**
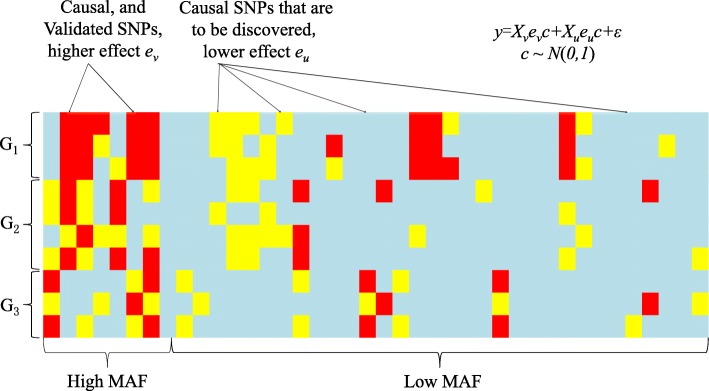
An illustration of the generation process of SNP array data. This figure shows the data is generated with three populations as an example

In addition, the known SNPs in our simulation have higher MAFs and larger coefficients than the SNPs to be discovered. More specifically, for a SNP *j*, if *j*∈*k* and *j*∈*v*, it simulates the SNP that is already known to be associated with the trait and it has coefficient *β*_*j*_=*e*_*v*_*c*_*j*_. On the other hand, if *j*∈*k* and *j*∈*u*, SNP *j* simulates the undiscovered associated SNP that has coefficient *β*_*j*_=*e*_*u*_*c*_*j*_. If *j*∉*k*, SNP *j* simulates a SNP that is not associated with the phenotype and has the coefficient *β*_*j*_=0*c*_*j*_=0. *c*_*j*_ is the base coefficient, sampled from a uniform distribution *U*(0,1). This simulation process is showed in Fig. 1.

We generate the associated phenotype **y** as **y**=**X***β*+*ε*, where *ε*∼*N*(0,1) is the natural noise. We further transform **y** into a binary phenotype with a Binomial sampling procedure with the probability of success achieved through feeding **y** into the inverse logit function.

Following [1], we conduct experiments with a variety of the settings with different combinations of MAFs (*m*_*u*_=0.005,0.01), coefficients (*e*_*u*_=5,10,25) of the SNPs to be discovered, and heritability (0.1,0.3,0.5,0.7) of the phenotype. For the known SNPs, we keep *m*_*v*_=0.1 and *e*_*v*_=50. We choose *n*=500, *p*=500000, and *k*=10 for the following experiments. For each configuration of the data, we repeat the experiments 10 times with different random seeds, and the reported result is based on the union of the results from all runs.

#### Evaluation

To conduct a fair comparison, we evaluate these models only regarding their ability to uncover the associated SNPs that are not already known to CS-LMM, as CS-LMM takes the known SNPs as a prior knowledge. For each method, we follow the convention to select the parameter *λ* (the weight of regularizer), which leads to the desired number of the selected variables (denoted as *K*) [3, 23]. This helps to avoid overly complex models, which tend to be selected by automatic measures such as cross validation, the Akaike information criterion (AIC), and the Bayesian information criterion (BIC) [24]. Moreover, it is known that the performance of parameter estimation and prediction are not directly coupled, e.g., as mentioned in [25] and the hyperparameter selected through cross-validation tend to report more false positives [3]. In our experiments, we select exactly *K*=*k* variables.

#### Results

**Fig. 2:**
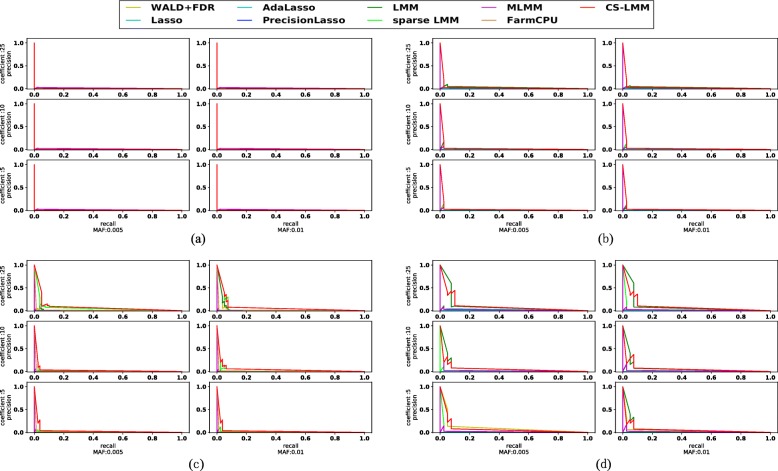
Simulation results of CS-LMM compared to other models in terms of the precision-recall curve. The x-axis is recall and y-axis is precision. This figure is split into four components based on the heritability. **a**: heritability is 0.1; **b** heritability is 0.3; **c** heritability is 0.5; **d** heritability is 0.7;

Figure 2 shows the precision-recall curve of CS-LMM compared to the Wald test, Lasso, Adaptive Lasso, Precision Lasso, LMM, sparse LMM, MLMM, and FarmCPU. The figure shows 24 experiments with three choices of coefficients (*e*_*u*_) across two choices of MAFs *m*_*u*_ of the SNPs to be discovered, and four choices of heritability. In particular, plots in Figure 2 represent MAFs and coefficients correspond to heritability 0.1 (a), 0.3 (b), 0.5(c), and 0.7(d).

Figure 2a represents the most challenging case since the heratibility is as small as 0.1. All the methods do not behave well in this setting, and MLMM seems to have tiny advantages over other methods. Figure 2b and c illustrate the more realistic cases with heritabilities set as 0.3 and 0.5. Within this set-up, we can see CS-LMM has clear advantages over other methods. Sparse LMM and vanilla LMM are also behaving well, but still inferior to CS-LMM. Figure 2d represents a simple scenario where the heritability is 0.7. In this setting, simpler univeriate testing methods, such as Wald and LMM, can also perform well, and CS-LMM behave roughly slightly shy of these univariate testing methods. In general, CS-LMM behave better than the competing methods in most settings of the experiments.

##### Other experiments

Other than the main experiment shown in Fig. 2, we have tested our methods in a larger range of choices of coefficients and MAF, tested the methods when we have different choices of *k*, and tested the methods under a larger number of samples. We also reported other other evaluation criteria including true positives, false positives and area under ROC (auROC) under a broader setting of the experiment. There more thorough tests are included in Additional file 1: Section 4–7.

Taken together, these results show that CS-LMM outperforms other competing existing approaches in most cases, in particular, in the settings when the heratibility is at an intermediate level. Notably, these are also the settings that resemble real life scenarios for complex human diseases, and thus demonstrating the necessity and promising usages of CS-LMM in the real life.

### Application to real data

#### Alcoholism study

We apply our method CS-LMM to the case-control GWAS data collected from subjects with and without alcoholism by The Center for Education and Drug Abuse Research (CEDAR) at the University of Pittsburgh. The data set consists of 383 individuals that include 305 subjects reported to be addicted to the consumption of alcohol through their lifetime. The data consists of 234 male subjects and 149 female subjects. The ages of these subjects range from 21 to 31. There are 519,138 genotyped SNPs in the data. The missing values are imputed as the mode of corresponding SNPs. To take the full advantage of our method, we collect the SNPs associated with alcoholism that are reported in GWAS Catalog [26] with p-values smaller than 1e-8 as the known SNPs to build in the CS-LMM model. The four SNPs we collect include: *rs1789891*, *rs7590720*, *rs2835872*, and *rs4478858*. With these known alcoholism-associated SNPs fed into CS-LMM, we run the model to uncover additional SNPs that have weaker associations with alcoholism.

We inquire 20 SNPs from the model, and CS-LMM returns 21 predicted SNPs when converges, including the 4 known SNPs we feed into the model as a prior knowledge, and thus the model discovers 17 alcoholism-associated SNPs. Table 1 lists the SNPs associated with alcoholism that are identified by CS-LMM. Since it is challenging to verify the reliability of these findings experimentally, we instead conduct a literature survey to find out whether the genes where these SNPs reside are linked to alcoholism or related disorders. Even though this type of “verification” may not provide conclusive evidence about the association between the identified SNPs and the disease, it can provide clues about whether the findings are worth further investigation.

**Table 1:** The top SNPs that CS-LMM identifies in an alcoholism study with four known associations

Rank	SNP	Chr	Chr Position	Est. Coe.	MAF	Gene	Disease [Literature]
1	rs1789891	4	99329262	4.2E3	0.15	*ADH1B*	ALC [27]
2	rs7590720	2	216033935	1.7E3	0.29	*PECR*	ALC [28]; AD [29]
3	rs2835872	21	37654970	1.5E3	0.25	*KCNJ6*	ALC [30]; DS [31]
4	rs4478858	1	31411078	1.4E3	0.44	*SERINC2*	ALC [32]
5	rs1789924	4	99353129	-2.2E-4	0.33	*ADH1C*	ALC [33]
6	rs698	4	99339632	-2.2E-4	0.33	*ADH1C*	ALC [33]
7	rs2851300	4	99358667	-2.2E-4	0.33		
8	rs10483038	21	37652469	-1.6E-4	0.25	*KCNJ6*	ALC [30]; DS [31]
9	rs1344694	2	216028914	-1.3E-4	0.32	*PECR*	ALC [28]; AD [29]
10	rs4147536	4	99317955	-7.6E-5	0.30	*ADH1B*	ALC [27]
11	rs12482570	21	37705475	-5.9E-5	0.28	*KCNJ6*	ALC [30]; DS [31]
12	rs857975	21	37629311	-5.8E-5	0.28	*KCNJ6*	ALC [30]; DS [31]
13	rs4147544	4	99213357	-5.7E-5	0.45	*ADH6*	ALC [34]
14	rs702860	21	37636327	-5.6E-5	0.26	*KCNJ6*	ALC [30]; DS [31]
15	rs2835853	21	37642590	-5.6E-5	0.26	*KCNJ6*	ALC [30]; DS [31]
16	rs717859	21	37640500	-5.6E-5	0.26	*KCNJ6*	ALC [30]; DS [31]
17	rs11499823	4	99353592	-5.6E-5	0.12	*ADH1C*	ALC [33]
18	rs2835910	21	37713604	-5.5E-5	0.30	*KCNJ6*	ALC [30]; DS [31]
19	rs4355398	4	99237168	-3.8E-5	0.25		
20	rs2187483	4	99212946	-9.7E-7	0.38	*ADH6*	ALC [34]
21	rs2835831	21	37614931	-6.9E-7	0.30		

The SNPs are ranked by the absolute values of their estimated coefficients. The first four SNPs with the largest coefficients in the upper panel are known SNPs that our model CS-LMM takes as prior knowledge. The rest SNPs in the lower panel are ones predicted by the model. The MAFs reported in the table are calculated using the case-control alcoholism GWAS data. The information of whether a SNP is located within a region of a gene is taken from the Database for Single Nucleotide Polymorphisms (dbSNP) [35], and listed in the ’Gene’ column. Abbreviations: ALC, Alcoholism; AD, Alzheimer’s Disease; DS, Down Syndrome; Est. Coe.: Estimated Coefficients. Note that the literature support may refer to how the genes that the corresponding SNPs reside in are related to the phenotype, instead of the SNPs themselves. See discussions in Section *Alcoholism Study* for details

Encouragingly, all the SNPs we discovered are linked to alcoholism, through the gene these SNPs reside in, in previously published results (shown in Table 1). For example, the 5^th^, the 6^th^, and the 17^th^ SNPs are within the region of the gene *ADH1C*, which encodes class I alcohol dehydrogenase, gamma subunit, a member of the alcohol dehydrogenase family. *ADH1C* has been shown to be associated with alcoholism in different populations [33]. Also, there are seven different SNPs residing within the region of *KCNJ6*, which encodes a member of the G protein-coupled inwardly-rectifying potassium channel. *KCNJ6* is also reported to be associated with alcoholism previously [30]. The 9^th^ SNP resides within the region of *PECR*. Interestingly, previous evidence shows that *PECR* is not only associated with alcoholism [28], but also plays some role in Alzheimer’s disease [29]. A previous study reported that the protein level of *PECR* is significantly altered in the cortical lipid rafts of the murine model of AD, compared to the control mice [29]. This result is consistent with a previous study suggesting associations between daily alcohol users and Alzheimer’s patients [36].

The 10^th^ SNP is within the region of *ADH1B*, which is also known to be related with alcoholism. The 13^th^ SNP and the 20^th^ SNP are within in the region of gene *ADH6*, which is also known as an alcohol dependence gene [34].

#### Alzheimer’s disease study

Encouraged by our results from the alcoholism association mapping, we take a step further to investigate whether there is a genetic link between alcoholism and AD. We apply our method to a late-onset AD dataset provided by Harvard Brain Tissue Resource Center and Merck Research Laboratories [37]. The genotype data was generated from 540 subjects, and consists of the measurements for about 500,000 SNPs. There are 82 male subjects and 87 female subjects. The gender of the rest patients are unidentified. There are 366 subjects diagnosed with AD. The average age of these subjects is 56. The missing values are imputed as the mode of the corresponding SNPs. We use the two SNPs, *rs2075650* (gene *APOE*) and *rs157580* (gene *TOMM40*) as a prior knowledge to build into CS-LMM. These two SNPs are reported to be associated with AD with p-value less than 1e-20 in GWAS Catalog [26]. We inquire the model for 20 SNPs that are associated with AD, and 22 SNPs are reported. The results are shown in Table 2. The reason that we use different thresholds (1e-20 for Alzheimer’s disease and 1e-8 for Alcoholism) to choose SNPs are prior knowledge is mainly due to the fact that Alzheimer’s disease is studied much more extensively than alcoholism in GWAS catalog, and p-values for SNPs that are reported to be associated with Alzheimer’s disease tend to be smaller than those for alcoholism. We verify our findings following the same logic presented in the previous section.

**Table 2:** The top SNPs that CS-LMM identifies in an AD study with two known associations

Rank	SNP	Chr	Chr Position	Est. Coe.	MAF	Gene	Disease [Literature]
1	rs2075650	19	44892362	0.21	0.18	*APOE*	AD [38]
2	rs157580	19	44892009	0.02	0.27	*TOMM40*	AD [39]
3	rs10027926	4	3412927	-8.3E-11	0.14	*RGS12*	SCZ [40]
4	rs12641989	4	3418113	-7.8E-11	0.14	*RGS12*	SCZ [40]
5	rs3088231	4	3420484	-7.5E-11	0.13	*RGS12*	SCZ [40]
6	rs10512523	17	69044919	5.2E-11	0.28	*ABCA9*	AD [41]
7	rs4076949	1	234066399	4.2E-11	0.18	*SLC35F3*	
8	rs874418	4	3440342	-3.9E-11	0.19	*HGFAC*	
9	rs6842419	4	3475572	-3.2E-11	0.16	*DOK7*	
10	rs16844383	4	3445516	-2.9E-11	0.21	*HGFAC*	
11	rs12131508	1	234017193	1.7E-11	0.17	*SLC35F3*	
12	rs12506821	4	3282833	-1.6E-11	0.16		
13	rs11485175	1	222437868	1.4E-11	0.23		
14	rs584507	10	6489788	1.2E-11	0.24	*PRKCQ*	
15	rs12563692	1	216818264	-1.2E-11	0.30	*ESRRG*	ALC [42, 43]
16	rs6446731	4	3283024	-1.1E-11	0.26		
17	rs7984051	13	70233817	-8.1E-12	0.25		
18	rs2327771	20	13295734	3.0E-12	0.29	*ISM1*	
19	rs7548651	1	234012812	2.4E-12	0.20	*SLC35F3*	
20	rs4330674	8	133209259	-1.2E-12	0.24	*WISP1*	
21	rs16885750	5	56578982	-8.1E-13	0.12	*C5orf67*	
22	rs938412	3	188571269	3.6E-13	0.31	*LPP*	

The SNPs are ranked by the absolute values of their estimated coefficients. The first two SNPs with largest coefficients are known SNPs the model takes as a prior knowledge. The rest are SNPs predicted by the model. The MAFs reported in the table are calculated using the AD GWAS data. The information of whether a SNP is located within a region of a gene is taken from the dbSNP. Abbreviations: ALC, Alchoholism; AD, Alzheimer’s Disease; SCZ, Schizophrenia; Est. Coe.: Estimated Coefficients. Note that the literature support may refer to how the genes that the corresponding SNPs reside in are related to the phenotype, instead of the SNPs themselves. See discussions in Section *Alzheimer’s Disease Study* for details.

Among the 19 SNPs associated with AD in Table 2, we found that the 6^th^ SNP within gene *ABCA9* is previously reported associated with AD [41], confirming again that our method CS-LMM can identify biologically meaningful variants. Also noticeably, the 15^th^ SNP resides within gene *ESRRG*, which encodes estrogen related receptor *γ*. Interestingly, evidence suggests that ERR *γ* plays key an role in alcohol-induced oxidative stress [42, 43]. This result also potentially verifies the existence of the pleiotropic effects between alcoholism and AD.

Since this short list of SNPs shows a promising application of CS-LMM, we also apply CS-LMM to identify a longer list of 200 SNPs for further studies. The longer list is reported in Additional file 1 (Section S2 and S3).

We also apply the competing existing methods to these two data sets, none of these methods identify a list of SNPs that are consistent with published results to the extent that CS-LMM achieves.

## Discussion

We developed a novel method: Constrained Sparse multi-locus Linear Mixed Model (CS-LMM) that conditions on the associations that have already been discovered to identify disease-associated SNPs with weaker signals. Our CS-LMM model accounts for polygenic effects as well as corrects for complex relatedness such as population structure, family structure and cryptic relatedness. Our simulation experiments show that CS-LMM outperforms other competing existing methods in terms of uncovering the variants with weaker signals in various settings which reflect real life scenarios for common and rare diseases. Interestingly, in the case of ’rare variants with weak coefficients’, which is categorized as the most challenging case in [1, 44], CS-LMM is superior to other competing methods. Our simulations also show that CS-LMM can particularly outperforms other methods consistently in terms of controlling false positives.

Furthermore, we apply CS-LMM to alcoholism and AD studies. For about top 20 SNPs associated with either alcoholism or AD that CS-LMM identifies, many of the SNPs reside within genes that were previously implicated in the corresponding diseases. Interestingly, our results further verify the pleiotropic effects between alcoholism and AD. The results indicate that two alcoholism-associated SNPs, *rs7590720* (previously known) and *rs1344694* (newly discovered), reside in PECR. The protein level of PECR was shown to be abnormally altered in a murine model of AD compared to the control mice, suggesting the involvement of PECR in the disease mechanism of AD. Similarly, our results also show that a novel AD-associated SNP, *rs12563692*, resides in ESRRG which encodes estrogen related receptor *γ*. Notably, ERR *γ* plays key an role in alcohol-induced oxidative stress and liver injury.

One interesting aspect regarding CS-LMM is about the three-phase learning algorithm we develop for estimating the parameters of the model. Two alternative strategies of learning the parameters are: 1) directly solving it as a convex optimization problem with explicit constrains; and 2) solving it as a standard Lasso with relaxation on the regularization on known associations. We tested these two algorithms in simulations, and our three-phase learning algorithm outperforms these two alternative strategies.

To tailor CS-LMM for case-control data or binary traits, a simple extension can be made that replaces the linear regression cost function with logistic regression cost function. Interestingly, our results indicates that CS-LMM works well with case-control data as it is (data not shown), without any extensions required. In fact, extending CS-LMM to logistic regression (or any other generalized linear models with a nontrivial link function) will affect the results adversely. For a generalized linear model, we believe CS-LMM will only function as desire when the link function is identity.

## Conclusions

In summary, we have proposed and developed a novel software tool, CS-LMM, for disease association mapping which takes into account genetic variants of known associations, polygenic effects, as well as population structure and complex relatedness. The results from our simulation experiments and real data analysis demonstrate that CS-LMM can be served as an effective tool for association studies for complex human diseases.

## Supplementary information

**Additional file 1** Supplementary of *Discovering Weaker Genetic Associations Guided by Known Associations, with Application to Alcoholism and Alzheimer’s Disease Studies*. The file has instructions of using the software and extra experimental results.

## Data Availability

The programs CS-LMM is available at https://github.com/HaohanWang/CS-LMM. The datasets used and analysed during the current study are available from the corresponding author on reasonable request.

## Abbreviations

AD: Alzheimer’s disease
CS-LMM: Constrained sparse multi-locus linear mixed model
GWAS: Genome wide association studies
LMM: Linear mixed model
MAF: Minor allele frequency
SNP: Single nucleotide polymorphism

## Footnotes

1 https://github.com/HaohanWang/CS-LMM
