# Supplementary material for: Discovering weaker genetic associations guided by known associations
Source: BMC Med Genomics. 2020 Feb 24;13(Suppl 3):19. doi: 10.1186/s12920-020-0667-4 (PMC7038505; doi:10.1186/s12920-020-0667-4)
Supplement: Supplementary file 1 — Additional file 1 Supplementary of Discovering Weaker Genetic Associations Guided by Known Associations, with Application to Alcoholism and Alzheimer’s Disease Studies. The file has instructions of using the software and extra experimental results. [file 12920_2020_667_MOESM1_ESM.pdf]

Supplementary of *Discovering weaker genetic associations guided by known associations*

Haohan Wang, Michael M. Vanyukov, Eric P. Xing, and Wei Wu

1 Instructions of Using the Software CS-LMM

Download and following the installation instruction at https://github.com/HaohanWang/CS-LMM.
One can also use CS-LMM software as a stand alone script without installation.
Run

```
python cslmm.py --help
```

for usage instructions as following:

```
Options:
-h, --help            show this help message and exit

Data Options:
  -t FILETYPE         choices of input file type
  -n FILENAME         name of the input file
  -v FILEVALIDATED    list of the validated markers

Model Options:
  --lambda=LMBD       the weight of the penalizer. If neither lambda or snum
                      is given, cross validation will be run.
  --snum=SNUM         the number of targeted variables the model selects. If
                      neither lambda or snum is given, cross validation will
                      be run.
  -s                  Stability selection
  -q                  Run in quiet mode
  -m                  Run without missing genotype imputation
```

Recommended Usage:

```
python cslmm.py -n data/mice.plink -v knownMarkers.txt --snum 20 -s
```

This command will first fit CS-LMM with the SNPs that are validated to be associated with the phenotype (stored in knownMarkers.txt, an id per line), and then run CS-LMM second phase to select 20 SNPs (together with the known ones) to be associated with the phenotype. The results will be stored in data/snps.132k.output.

Naive Usage

```
python cslmm.py -n data/mice.plink
```

This command does not require the user to specify any arguments. CS-LMM will first conduct Wald test to identify the most significantly associated SNPs and then fit the rest SNPs with the residue of fitting these SNPs. CS-LMM will also perform cross validation to select the appropriate regularization weight.

2 Longer List of Discovered Alcoholism SNPs

We run the our method with the same setting in the main paper except querying for a longer list (200 SNPs) of potential associated SNPs. The method converges with 155 SNPs. The MAF is calculated with the experimental data set.

Rank	SNP	Chr	Chr Position	Allele	MAF	GENE
1	rs1789891	4	99329261	A/C	0.1593	ADH1B
2	rs7590720	2	216033934	A/G	0.2859	PECR
3	rs2835872	21	37654969	A/G	0.252	KCNJ6
4	rs4478858	1	31411077	A/G	0.4413	SERINC2
5	rs2851300	4	99358666	C/T	0.3303	ADH1C
6	rs698	4	99339631	A/G/T	0.3303	ADH1C
7	rs1789924	4	99353128	C/T	0.3303	
8	rs10483038	21	37652468	C/T	0.252	KCNJ6
9	rs1344694	2	216028913	G/T	0.3185	PECR
10	rs4147536	4	99317954	G/T	0.2977	ADH1B
11	rs12482570	21	37705474	A/G	0.2846	KCNJ6
12	rs857975	21	37629310	A/C	0.2833	KCNJ6
13	rs4147544	4	99213356	A/C	0.4504	ADH6
14	rs2835853	21	37642589	A/C	0.2572	KCNJ6
15	rs717859	21	37640499	A/G	0.2572	KCNJ6
16	rs702860	21	37636326	A/G	0.2572	KCNJ6
17	rs11499823	4	99353591	C/T	0.1175	ADH1C
18	rs2835910	21	37713603	C/T	0.2963	KCNJ6
19	rs4355398	4	99237167	A/G	0.2454	
20	rs2187483	4	99212945	A/C	0.3838	ADH6
21	rs2835831	21	37614930	C/T	0.299	
22	rs702859	21	37625398	C/T	0.2728	KCNJ6
23	rs705648	2	216069068	C/T	0.2572	PECR
24	rs857978	21	37625823	C/T	0.2924	KCNJ6
25	rs6833176	4	99210005	C/G	0.3903	ADH6
26	rs1709835	21	37665333	C/T	0.3943	KCNJ6
27	rs10047085	1	31429570	A/G	0.4034	SERINC2
28	rs934153	2	216025780	A/G	0.3094	
29	rs1399591	21	37681653	A/G	0.4896	KCNJ6
30	rs4371638	4	76519113	C/T	0.4086	SHROOM3
31	rs11576621	1	31322612	A/G	0.4099	ZCCHC17
32	rs1566961	1	31299602	C/T	0.4099	ZCCHC17
33	rs1159918	4	99321851	G/T	0.4726	ADH1B
34	rs4949372	1	31266009	A/G	0.406	SNRNP40
35	rs12121165	1	31252694	A/G	0.406	
36	rs7523438	1	31315465	A/G	0.3956	ZCCHC17
37	rs858008	21	37693326	C/T	0.4634	KCNJ6
38	rs1229976	4	99280920	C/T	0.1932	ADH1A
39	rs4147545	4	99207595	A/G	0.3995	ADH6
40	rs3857224	4	99208527	C/T	0.3903	ADH6
41	rs1372679	4	99383372	C/T	0.0927	
42	rs12562824	1	31382034	A/G	0.2141	
43	rs1372680	4	99383387	A/C	0.107	
44	rs1614972	4	99336997	C/T	0.3812	ADH1C
45	rs4949391	1	31360858	G/T	0.2128	FABP3
46	rs1995585	1	31382626	A/G	0.2128	UKN
47	rs4147547	4	99206153	A/C	0.342	ADH6
48	rs6663779	1	31362679	C/T	0.201	FABP3
49	rs777057	1	105548693	A/C	0.4399	UKN
50	rs6719499	2	79966259	A/G	0.2924	CTNNA2

Rank	SNP	Chr	Chr Position	Allele	MAF	GENE
51	rs17548312	2	216086523	A/G	0.1997	TMEM169
52	rs9621631	22	33071070	C/T	0.3538	
53	rs1316509	1	31507154	C/T	0.4778	
54	rs2071122	X	153786451	A/G	0.3238	IDH3G
55	rs7585163	2	205028448	A/G/T	0.4948	PARD3B
56	rs4763707	12	11561719	A/G	0.4452	
57	rs1006841	17	66637120	A/C/T	0.4465	PRKCA
58	rs6425766	1	31542070	A/G	0.3956	
59	rs877447	17	66630414	A/G	0.4517	PRKCA
60	rs4773200	13	110503673	C/T	0.4269	COL4A2
61	rs3828541	4	99121110	A/G	0.3956	
62	rs2835886	21	37668039	C/T	0.483	KCNJ6
63	rs1008542	21	37668258	A/G	0.4843	KCNJ6
64	rs10521391	X	79267137	C/T	0.3003	
65	rs4813720	20	4816762	A/G	0.3655	RASSF2
66	rs4819853	22	20012236	A/G	0.3225	ARVCF
67	rs9389206	6	134704614	A/G	0.3525	
68	rs10914420	1	31511748	A/G	0.4347	
69	rs17670486	3	114339565	C/T	0.3486	ZBTB20
70	rs7061782	X	32884194	C/T	0.3982	DMD
71	rs6139039	20	3230045	A/G	0.3747	SLC4A11
72	rs7324140	13	105281349	C/T	0.2911	
73	rs694372	18	351113	A/C	0.4099	COLEC12
74	rs6435660	2	211707213	C/T	0.4843	ERBB4
75	rs2153275	1	31523051	C/T	0.4778	
76	rs6659255	1	31396821	C/T	0.1606	
77	rs4828534	X	16842789	A/G	0.4373	TXLNG
78	rs2835906	21	37710501	C/T	0.4922	KCNJ6
79	rs10493047	1	31268929	C/T	0.1462	SNRNP40
80	rs2127514	X	16868457	A/C	0.3708	RBBP7
81	rs8142672	22	33073044	C/T	0.2676	
82	rs2702235	X	16244450	A/G	0.406	
83	rs5933262	X	133075157	C/T	0.3943	USP26
84	rs12396431	X	44447517	A/G	0.2115	
85	rs6548551	3	78293141	A/G	0.3799	
86	rs2835908	21	37712186	A/C	0.3225	KCNJ6
87	rs4699748	4	99400285	A/G	0.1397	
88	rs3851675	12	131604382	A/G	0.4191	
89	rs3850163	X	28427519	G/T	0.2781	
90	rs6567588	X	3701482	A/G	0.3368	PRKX
91	rs17409	X	144907362	A/C	0.3916	
92	rs10500899	11	21173899	C/T	0.3838	NELL1
93	rs400532	13	110502283	A/C	0.3512	COL4A2
94	rs726102	11	10262430	C/T	0.4843	SBF2
95	rs11042702	11	10266440	A/G	0.4817	SBF2
96	rs6425760	1	31522526	A/G	0.4504	
97	rs2406669	X	33349241	G/T	0.4151	
98	rs7544003	1	31216240	C/T	0.4974	NKAIN1
99	rs7520468	1	31215964	A/G	0.4974	NKAIN1
100	rs542255	2	79969609	G/T	0.1997	CTNNA2

Rank	SNP	Chr	Chr Position	Allele	MAF	GENE
101	rs3915250	X	16305241	A/G	0.3629	
102	rs651595	X	123427505	A/G	0.4478	GRIA3
103	rs7110312	11	10121565	A/G	0.483	SBF2
104	rs12431748	14	97700062	A/G	0.3655	
105	rs4939881	18	49635362	C/T	0.436	
106	rs951317	3	106839534	G/T	0.3916	
107	rs3015363	13	105251988	A/G	0.4961	
108	rs4597056	11	10269375	C/T	0.4804	SBF2
109	rs6657902	1	31525181	A/G	0.4869	
110	rs1482455	6	63987927	C/T	0.3668	EYS
111	rs4938648	11	111464549	C/T	0.3016	BTG4
112	rs5928243	X	33351227	C/T	0.3838	
113	rs1945321	11	21174633	A/C	0.3799	NELL1
114	rs7912438	10	1832159	C/T	0.4883	
115	rs10798852	1	31448483	A/G	0.3564	
116	rs10500898	11	21173805	C/T	0.4008	NELL1
117	rs951545	1	31371817	C/T	0.1632	FABP3
118	rs7661978	4	99363041	C/T	0.2389	
119	rs4827630	X	145221632	G/T	0.4961	
120	rs5919813	X	145219102	C/T	0.4961	
121	rs4364664	8	70092861	C/T	0.4569	
122	rs6672056	1	31215263	C/T	0.423	NKAIN1
123	rs12319239	12	131608059	G/T	0.4883	
124	rs10833470	11	21168479	A/C	0.3642	NELL1
125	rs1038316	6	64002975	A/G	0.3681	EYS
126	rs2800881	1	111591287	C/T	0.3394	RAP1A
127	rs4806146	19	35380084	G/T	0.4869	
128	rs7940204	11	21170582	C/T	0.376	NELL1
129	rs8081512	17	66712747	A/G	0.4373	PRKCA
130	rs1884299	X	47310637	C/T	0.2833	
131	rs6760872	2	205066041	C/T	0.2924	PARD3B
132	rs5925062	X	151843727	C/T	0.4321	
133	rs4462057	X	132718462	A/G	0.4648	HS6ST2
134	rs2035256	5	80700812	A/G	0.4465	MSH3
135	rs6475768	9	23953635	C/T	0.4452	
136	rs440424	X	105618188	A/G	0.4178	IL1RAPL2
137	rs828704	2	216128887	A/C	0.1789	XRCC5
138	rs1955161	4	55788079	C/T	0.4204	
139	rs10057578	5	164986585	C/T	0.3943	
140	rs3908999	13	107700502	C/T	0.3473	FAM155A
141	rs1105289	11	132770460	C/T	0.3916	OPCML
142	rs1042026	4	99307308	A/G	0.2467	ADH1B
143	rs10500896	11	21169469	C/T	0.376	NELL1
144	rs6680246	1	31214811	A/G	0.4974	NKAIN1
145	rs9344218	6	81681242	G/T	0.4856	
146	rs12072863	1	83362589	A/G	0.4687	
147	rs4519409	18	25142112	A/C	0.3551	ZNF521
148	rs5962542	X	105536509	A/G	0.4883	IL1RAPL2
149	rs4661617	1	15267169	C/T	0.3225	FHAD1
150	rs10955035	8	37881097	A/G	0.4504	RAB11FIP1
151	rs1106431	1	18038890	A/G/T	0.2689	
152	rs6936438	6	64009770	C/T	0.3877	EYS
153	rs17457845	1	105655139	A/C	0.483	
154	rs1736646	X	79340998	C/T	0.4112	
155	rs9873496	3	106314579	C/T	0.4008	

3 Longer List of Discovered Alzheimer's SNPs

We also run the our method for 200 potential associated SNPs in Alzheimer's disease. The method reports 161 SNPs. The MAF is calculated with the experimental data set. The last column indicates whether this SNP has been reported in GWAS Catalog previously.

Rank	SNP	Chr	Chr Position	Allele	MAF	GENE
1	rs2075650	19	44892361	A/G	0.1824	APOE
2	rs157580	19	44892008	A/G	0.2657	TOMM40
3	rs10027926	4	3412926	A/G	0.1407	RGS12
4	rs12641989	4	3418112	A/G	0.137	RGS12
5	rs3088231	4	3420483	A/G	0.1324	RGS12
6	rs10512523	17	69044918	A/G	0.2806	ABCA9
7	rs4076949	1	234066398	C/T	0.1787	SLC35F3
8	rs874418	4	3440341	C/T	0.1889	HGFAC
9	rs6842419	4	3475571	A/G	0.1602	DOK7
10	rs16844383	4	3445515	C/T	0.213	HGFAC
11	rs12131508	1	234017192	A/G	0.1676	SLC35F3
12	rs12506821	4	3282832	C/T	0.1556	
13	rs11485175	1	222437867	C/T	0.2296	
14	rs584507	10	6489787	C/T	0.2398	PRKCQ
15	rs12563692	1	216818263	C/T	0.3037	ESRRG
16	rs6446731	4	3283023	A/G	0.2639	
17	rs7984051	13	70233816	A/G	0.2481	
18	rs2327771	20	13295733	A/G	0.2898	ISM1
19	rs7548651	1	234012811	G/T	0.2	SLC35F3
20	rs4330674	8	133209258	C/T	0.237	WISP1
21	rs16885750	5	56578981	C/T	0.1167	C5orf67
22	rs938412	3	188571268	C/T	0.3093	LPP
23	rs4421636	1	76319707	C/T	0.2556	ST6GALNAC3
24	rs10818118	9	118163681	C/T	0.25	
25	rs11754527	6	3819181	A/G	0.2907	
26	rs10238983	7	34859960	C/T	0.2213	NPSR1
27	rs7109505	11	36267201	A/C	0.3259	
28	rs7938549	11	41542213	A/G	0.2694	
29	rs2063370	7	118641792	C/T	0.2546	
30	rs9485288	6	148377370	C/T	0.2722	SASH1
31	rs743067	21	13797857	A/G	0.2333	
32	rs11771057	7	118635827	C/T	0.2556	
33	rs6431431	2	236139534	C/T	0.3259	
34	rs993900	7	85376623	A/G	0.212	
35	rs9636594	21	14756349	G/T	0.262	
36	rs155106	2	181486624	A/C	0.2546	ITGA4
37	rs2618699	1	237502558	C/T	0.2926	RYR2
38	rs7793626	7	89158933	C/T	0.2528	ZNF804B
39	rs1361714	1	85494472	A/G	0.2546	DDAH1
40	rs9675039	17	25367	A/G	0.2694	METRNL
41	rs2843964	21	37062324	C/T	0.2556	
42	rs4922579	11	32114889	C/T	0.2519	
43	rs1557092	1	225768216	C/T	0.2741	
44	rs241430	6	4234296	A/G	0.2694	TAP2
45	rs10871741	18	57168327	A/G	0.3	
46	rs13228264	7	89183079	A/G	0.2528	ZNF804B
47	rs2822892	21	14749815	A/G	0.263	
48	rs9493157	6	131968016	C/T	0.2065	
49	rs2816764	14	49374888	A/G	0.1861	
50	rs11868097	17	69065756	A/G	0.2685	ABCA6

Rank	SNP	Chr	Chr Position	Allele	MAF	GENE
51	rs957628	12	107585975	A/G	0.2028	BTBD11
52	rs8019294	14	49408385	C/T	0.188	
53	rs8008868	14	49407040	A/G	0.188	
54	rs2296973	13	46892645	G/T	0.2972	HTR2A
55	rs10091244	8	55931370	G/T	0.2944	LYN
56	rs10838176	11	43810001	A/G	0.2731	HSD17B12
57	rs7145145	14	36110723	A/G	0.2741	
58	rs16844364	4	3436915	A/G	0.2185	RGS12
59	rs6507451	18	41938555	C/G/T	0.3037	
60	rs6972870	7	118682257	A/G	0.2491	
61	rs7694863	4	12538363	A/G	0.1981	
62	rs12812221	12	56925551	A/C	0.1583	SDR9C7
63	rs16863792	2	176417226	A/G	0.2389	
64	rs6129693	20	40852633	A/C	0.2667	
65	rs6539349	12	107587838	G/T	0.2056	BTBD11
66	rs562850	7	105705670	A/G	0.1648	ATXN7L1
67	rs963281	7	85380430	A/G	0.2102	
68	rs2833249	21	31064490	C/T	0.3056	
69	rs7689954	4	40449376	C/T	0.2574	RBM47
70	rs937737	10	113410345	A/C	0.2296	
71	rs9862823	3	179747969	C/T	0.263	USP13
72	rs2805425	1	237507256	A/G	0.2722	RYR2
73	rs6601501	8	10678315	C/T	0.1954	C8orf74
74	rs7668784	4	3337310	G/T	0.2167	RGS12
75	rs6851677	4	12550828	A/G	0.1991	
76	rs7965820	12	3122980	G/T	0.2046	TSPAN9
77	rs1353825	3	1296574	C/T	0.238	CNTN6
78	rs7042155	9	16056228	A/C	0.2472	CCDC171
79	rs9648813	7	144437557	C/T	0.2306	
80	rs6025871	20	57890561	C/T	0.2926	
81	rs2921325	11	70801857	C/T	0.3278	SHANK2
82	rs9948913	18	41934454	C/T	0.3028	
83	rs1035285	12	30960528	A/G	0.2963	TSPAN11
84	rs9288486	2	214064740	C/T	0.2981	SPAG16
85	rs2135411	3	30073622	C/T	0.2731	
86	rs997758	9	90052137	A/G	0.2463	
87	rs803410	6	150832368	C/T	0.2648	PLEKHG1
88	rs4121647	18	42115421	C/T	0.3009	
89	rs6774841	3	40576047	C/T	0.2769	
90	rs9529708	13	70237755	A/G	0.2463	
91	rs12324301	15	100246287	A/G	0.1685	ADAMTS17
92	rs701851	10	93417944	C/T	0.2	MYOF
93	rs1887826	13	70232417	C/T	0.2352	
94	rs2381181	9	4938231	C/T	0.2056	
95	rs11970980	7	87081035	A/C	0.2824	
96	rs6950509	7	85412696	C/T	0.2074	
97	rs2067613	3	176189553	A/G	0.2806	
98	rs12628419	22	43318248	C/T	0.3556	SCUBE1
99	rs2225163	9	14592181	A/C	0.2611	ZDHHC21
100	rs10829413	10	128470056	A/G	0.2806	

Rank	SNP	Chr	Chr Position	Allele	MAF	GENE
101	rs2959025	8	104467951	A/G	0.2907	DPYS
102	rs2049974	22	36546275	A/G	0.2963	
103	rs6802186	3	131715414	C/T	0.3241	CPNE4
104	rs9542210	13	70191895	C/T	0.2435	
105	rs1999749	10	118452644	G/T	0.2537	
106	rs13122924	4	179412951	A/G	0.2907	
107	rs778865	7	118707579	C/T	0.2546	
108	rs4832647	2	16524127	A/G	0.2852	
109	rs2306238	1	237550802	C/T	0.1954	RYR2
110	rs3102817	7	69079064	G/T	0.2796	
111	rs4628411	X	23212530	C/T	0.3852	PTCHD1-AS
112	rs794776	6	15288523	A/G	0.4	JARID2
113	rs4790859	17	1994525	A/G	0.2333	RTN4RL1
114	rs4868126	5	171856464	G/T	0.2639	
115	rs9321247	6	130685553	A/G	0.2546	
116	rs9532412	13	39657777	C/T	0.2935	COG6
117	rs6933963	6	124749771	A/C	0.2491	NKAIN2
118	rs1557627	22	19689683	C/T	0.2398	
119	rs7824502	8	82033529	A/G	0.2991	
120	rs1241079	18	35550963	C/T	0.2704	
121	rs10930731	2	176437961	A/G	0.25	
122	rs1443363	9	90068632	C/T	0.2481	
123	rs1571447	9	90066863	A/G	0.2481	
124	rs9514356	13	105079562	C/T	0.3676	
125	rs236476	6	36722929	A/G	0.2926	RAB44
126	rs241433	6	4230271	G/T	0.2657	TAP2
127	rs2176507	1	223420891	C/T	0.2861	
128	rs17712902	7	39427027	C/T	0.0954	POU6F2
129	rs4894505	3	176203095	G/T	0.2796	
130	rs9290704	3	102441660	G/T	0.2667	ZPLD1
131	rs11638250	15	35468738	C/T	0.2407	DPH6
132	rs4391047	4	12581256	C/T	0.2009	
133	rs1145768	6	90860827	C/T	0.2583	
134	rs11033542	11	36303411	A/G	0.2991	PRR5L
135	rs12040106	1	223434046	G/T	0.2861	
136	rs12046563	1	42671608	A/G	0.2333	PPIH
137	rs2833251	21	31067781	A/G	0.3074	
138	rs208807	20	38870495	A/G	0.2593	PPP1R16B
139	rs925351	7	118660335	A/G	0.2528	
140	rs9297040	6	17583603	C/T	0.237	
141	rs4974035	3	40558900	A/G	0.2704	
142	rs803401	6	150838493	A/G	0.2759	PLEKHG1
143	rs7583955	2	230168571	A/C	0.2389	SP110
144	rs12087818	1	233219782	C/T	0.2991	PCNX2
145	rs2997977	1	237506067	A/G	0.2787	RYR2
146	rs7146832	14	49420604	A/C	0.2056	
147	rs9573412	13	74542953	C/T	0.3111	
148	rs236137	20	5909498	C/T	0.2852	CHGB
149	rs17163831	1	223353650	A/G	0.2898	SUSD4
150	rs2032088	21	37105029	A/G	0.2769	TTC3

Rank In Catalog	SNP	Chr	Chr Position	Allele	MAF	GENE
151	rs2236620	22	116884	C/T	0.2704	SLC2A11
152	rs2805468	1	237557810	A/G	0.2426	RYR2
153	rs1941526	18	42072974	A/G	0.3	PIK3C3
154	rs9598129	13	33806509	C/T	0.1481	
155	rs10910030	1	2104244	C/T	0.2972	PRKCZ
156	rs2689753	7	118606625	C/T	0.2528	
157	rs12883776	14	56264412	A/G	0.2657	PELI2
158	rs6781787	3	22120073	A/C	0.2241	ZNF385D
159	rs17271519	3	1294712	A/G	0.2657	CNTN6
160	rs10111769	8	21101244	C/T	0.2815	
161	rs15653	2	16550241	A/G	0.2944	FAM49A

Table S8: The AUC scores of the Precision-recall curves in the main manuscript

h	MAF	Coef.	Wald	Lasso	AL	PL	LMM	SparseLMM	MLMM	FarmCPU	CS-LMM
0.1	0.005	5	0	0	0	0	0	**0.012**	**0.012**	0	0
	0.005	10	0	0	0	0	0	**0.012**	**0.012**	0	0
	0.005	25	0	0	0	0	0	**0.012**	**0.012**	0	0
	0.01	5	0	0	0	0	0	**0.012**	**0.012**	0	0
	0.01	10	0	0	0	0	0	**0.012**	**0.012**	0	0
	0.01	25	0	0	0	0	0	**0.012**	**0.012**	0	0
0.3	0.005	5	0	0	0	0	0.014	0.013	0.013	0	**0.027**
	0.005	10	0	0	0	0	0.013	0.013	0.013	0	**0.027**
	0.005	25	0	0	0.022	0	**0.040**	0.012	0.012	0	0.028
	0.01	5	0	0	0	0	0.012	0.013	0.013	0	**0.028**
	0.01	10	0	0	0	0	0.014	0.013	0.012	0	**0.028**
	0.01	25	0	0	0.012	0	**0.038**	0.012	0.012	0	0.029
0.5	0.005	5	0.007	0	0	0	0.008	0.019	0.007	0.013	**0.036**
	0.005	10	0.007	0	0	0	0.018	0.027	0.007	0.013	**0.033**
	0.005	25	0.008	0	0	0	0.031	0.062	0.007	0.013	**0.084**
	0.01	5	0.007	0	0	0	0.008	0.020	0.007	0.013	**0.036**
	0.01	10	0.007	0	0	0	0.017	0.045	0.007	0.013	**0.049**
	0.01	25	0.027	0	0	0	0.035	0.076	0.007	0.013	**0.077**
0.7	0.005	5	0.101	0	0	0	0.077	0.027	0.013	0	**0.078**
	0.005	10	0	0	0	0	**0.078**	0.013	0.027	0	0.062
	0.005	25	0	0	0.023	0	**0.105**	0.013	0.013	0	0.103
	0.01	5	0.045	0	0	0	**0.075**	0.027	0.014	0	0.061
	0.01	10	0	0	0	0	**0.072**	0.013	0.014	0	0.065
	0.01	25	0	0	0.012	0	0.097	0.027	0.013	0	**0.099**

4 AUC scores of the main result

We report the AUC scores of the the Precision-recall curves in the main manuscript to have a clearer understanding of the comparison of these methods in Table S8. The results demonstrate the strength of our method when heritability is intermediate, consistent with the main message delivered in our manuscript.

5 Simulation over a Larger Range of Settings.

Figure S1 shows the result of a larger range of simulation settings. Figure S2 shows the result when we tune the parameters by mis-specifying the number of associated SNPs as half of the actual number of associated SNPs and Figure S3 shows the result when we mis-specify that number as twice the number of associated SNPs. These figures show that directly tuning the parameter based on the number of associated SNPs selected is a valid and stable manner. More importantly, these figures show that CS-LMM is a promising method even the number of associated SNPs to select is mis-specified.

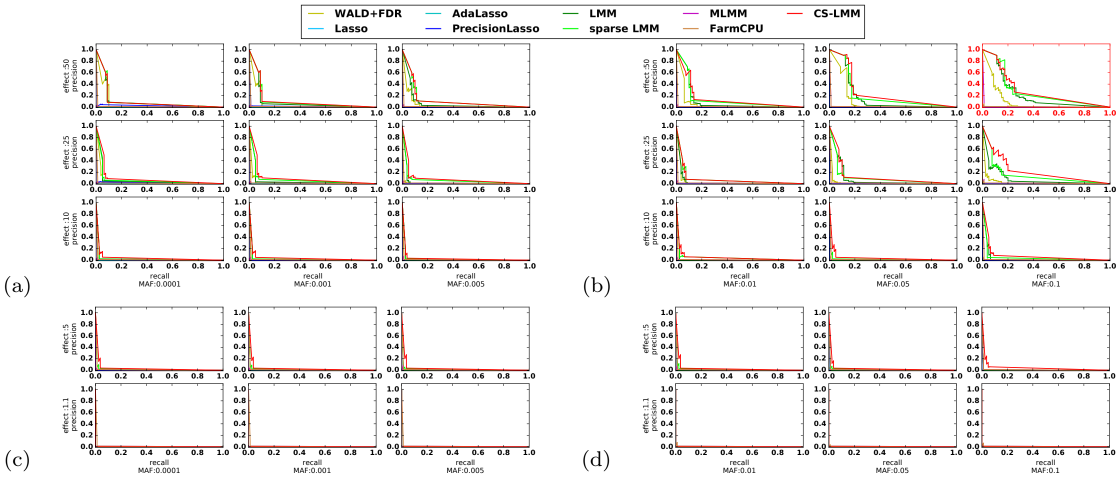

Figure S1: Simulation results of CS-LMM compared to other models in terms of the precision-recall curve when we query the models for $K = 2k$ SNPs. The x-axis is recall and y-axis is precision.

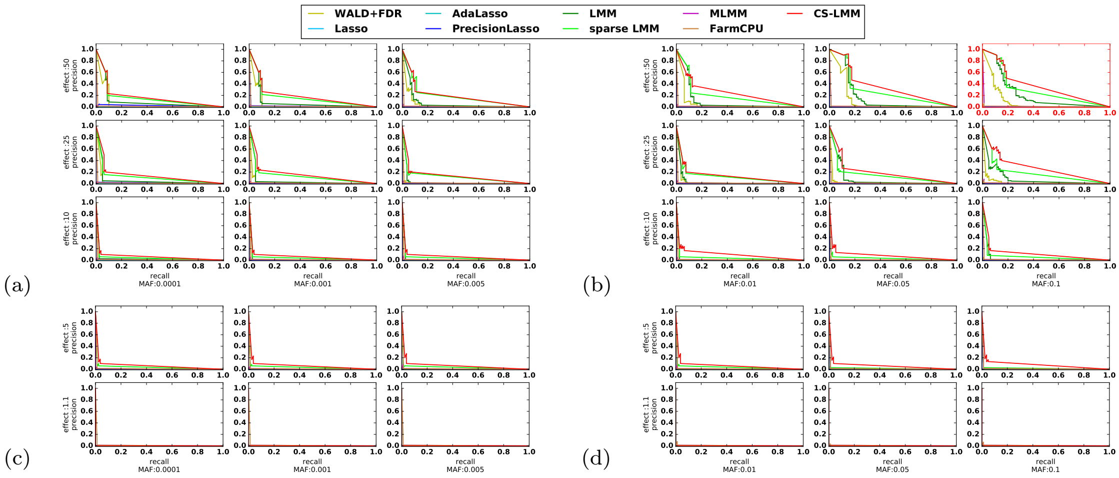

Figure S2: Simulation results of CS-LMM compared to other models in terms of the precision-recall curve when we query the models for $K = k/2$ SNPs. The x-axis is recall and y-axis is precision.

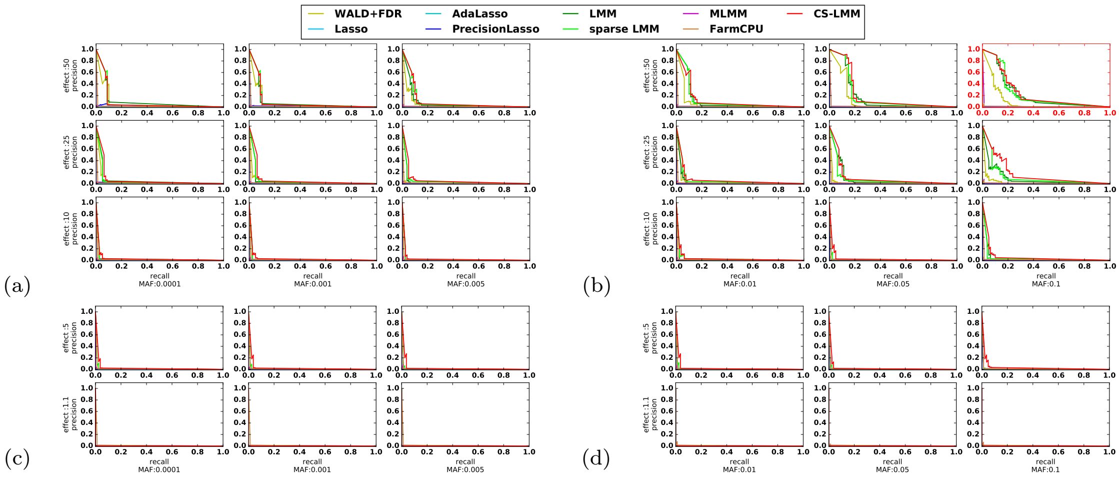

Figure S3: Simulation results of CS-LMM compared to other models in terms of the precision-recall curve when we query the models for $K = 2k$ SNPs. The x-axis is recall and y-axis is precision.

6 Evaluation of Other Configurations (Different Number of Samples and Different Number of Associated SNPs)

We test the performance of our method compared to other models with different configurations of k ($k \in \{5, 10, 50\}$) and n ($n \in \{250, 500, 1000\}$) in the situations where coefficient $e = \{5, 25\}$ and MAF $m = \{0.005, 0.01\}$. Fig. S4 and Fig. S5 show that our method is superior to other methods in most of these different configurations.

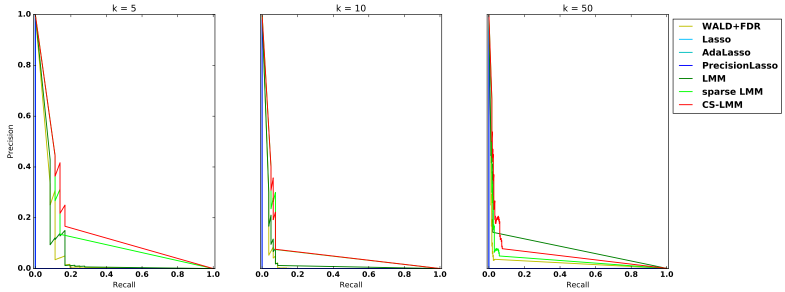

(a) k varies when $e = 25$ and $m = 0.01$

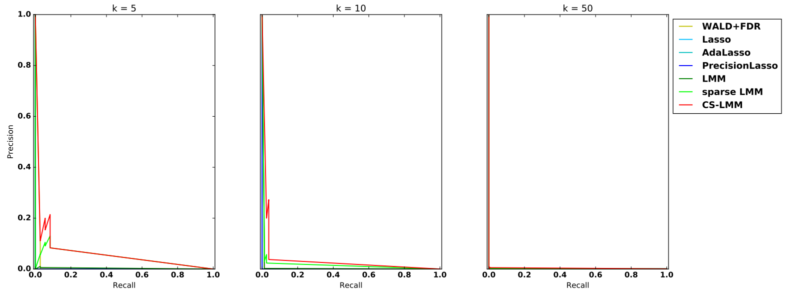

(b) k varies when $e = 5$ and $m = 0.005$

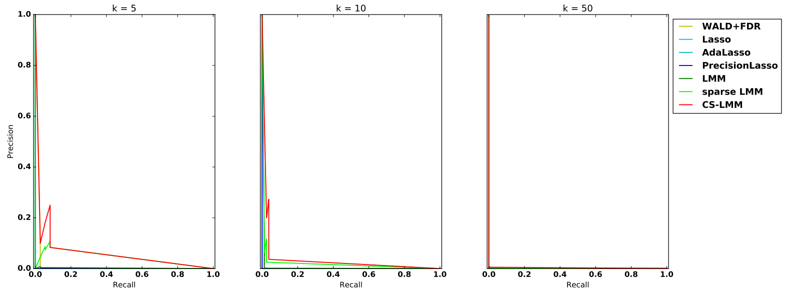

(c) k varies when $e = 5$ and $m = 0.01$

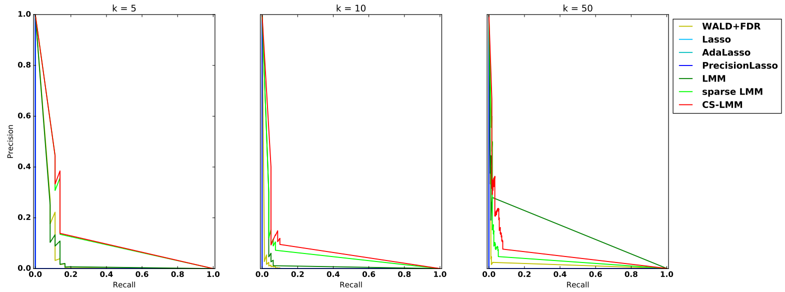

(d) k varies when $e = 25$ and $m = 0.005$

Figure S4: Evaluation of CS-LMM compared to other methods under different configurations of k (k varies in $\{5, 10, 50\}$ for different e and m). The x-axis is recall and y-axis is precision.

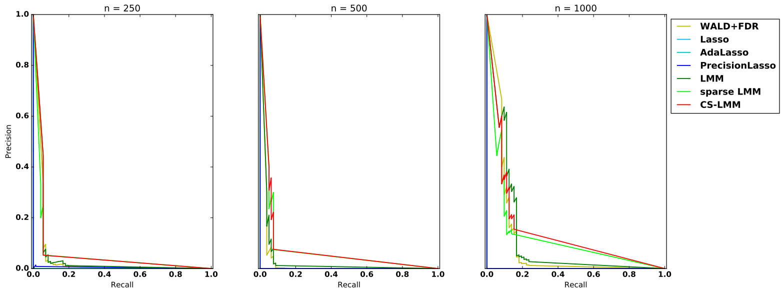

(a) n varies when $e = 25$ and $m = 0.01$

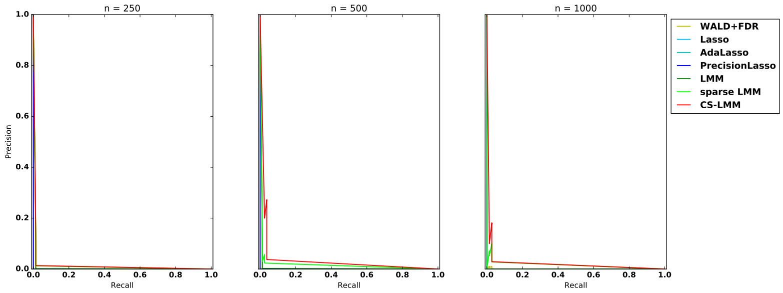

(b) n varies when $e = 5$ and $m = 0.005$

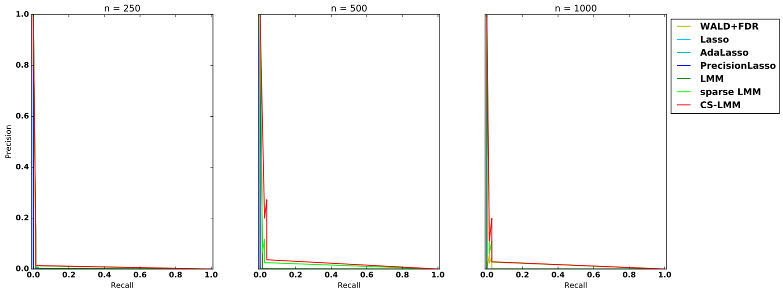

(c) n varies when $e = 5$ and $m = 0.01$

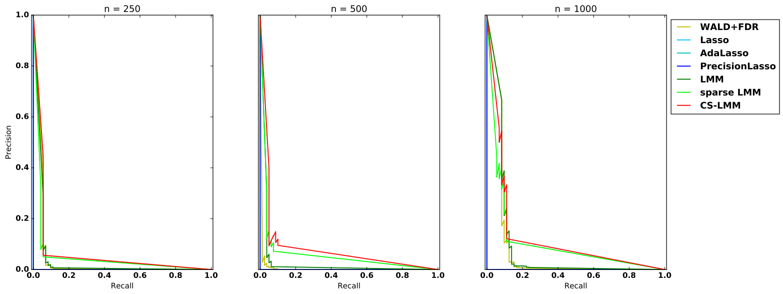

(d) n varies when $e = 25$ and $m = 0.005$

Figure S5: Evaluation of CS-LMM compared to other methods under different configurations of n (n varies in $\{250, 500, 1000\}$ for different e and m). The x-axis is recall and y-axis is precision.

7 Evaluation of Other Configurations (Large Scale Data)

We also tested the performance of CS-LMM in comparison with other methods with large scale data (when there are 10000 samples). As shown in Fig. S6, LMM based methods behave comparably with CS-LMM. CS-LMM still has slight advantages in most cases.

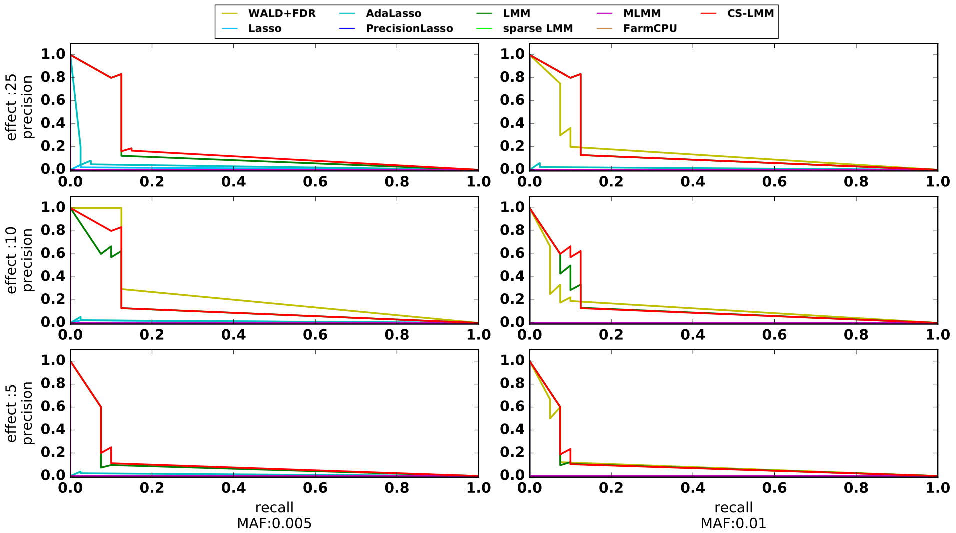

Figure S6: Simulation results of CS-LMM compared to other models in terms of the precision-recall curve when data there are 10000 samples.

8 Other Evaluation Metrics Applied to the Methods

We also evaluate CS-LMM and the competing methods using true positives, false positives and area under ROC (auROC) curves for the simulation experiments illustrated in Figure 2 in the main text. The results are shown in Table 8-Table S12. Note that these methods are evaluated only with unknown SNPs. This is a challenging task considering that other SNPs with stronger coefficients may mask the signals of the unknown SNPs, which explains the reason why many methods, in particular the Lasso and its variants, only perform slightly better than chance in terms of the auROC score.

By looking at the 'auROC' column, we can see that CS-LMM has an advantage over the other methods particularly when the cases are more challenging: rare variants with strong effects (Table 8), rare variants with small effects (Table S11), common variants with small effects (Table S12), respectively). As the coefficients and MAFs increase (Table S11 representing the unusual scenario of common variants with large effects), the unknown SNPs have as strong signals as the known SNPs, the Wald test and LMM become comparable and perform even better than CS-LMM in terms of the auROC score. However, it can be seen from the 'FP' column, both the Wald test with the FDR control and LMM report too many false positives, making these methods unrealistic in the real life applications. Also, as shown in the 'FP' column, CS-LMM outperforms other methods in terms of controlling false positives in most cases. We also notice that MLMM and FarmCPU also behave well in controlling false positives, especially when the signals are very small.

Table S9: The scenario of rare variants with strong effects.

MAF	coefficient	model	TP	FP	auROC
		WALD+FDR	**0.9**	1413.4	0.543
		Lasso	0.0	10.0	0.5
		AdaLasso	0.0	9.7	0.5
		PrecisionLasso	0.4	9.0	0.525
0.0001	50	LMM+FDR	0.8	8.5	**0.55**
		sparse LMM	0.7	7.7	0.544
		MLMM	0.1	7.9	0.506
		FarmCPU	0.1	7.9	0.506
		CS-LMM	0.7	**7.6**	0.544
		WALD+FDR	**1.0**	1617.8	0.548
		Lasso	0.0	9.9	0.5
		AdaLasso	0.0	9.8	0.5
		PrecisionLasso	0.2	9.4	0.512
0.001	50	LMM+FDR	0.9	14.4	**0.556**
		sparse LMM	0.8	8.2	0.55
		MLMM	0.1	7.9	0.506
		FarmCPU	0.1	7.9	0.506
		CS-LMM	0.8	**7.3**	0.55
		WALD+FDR	**1.7**	2099.4	**0.588**
		Lasso	0.0	9.9	0.5
		AdaLasso	0.0	9.8	0.5
		PrecisionLasso	0.0	9.5	0.5
0.005	50	LMM+FDR	1.2	38.1	0.575
		sparse LMM	0.9	**7.4**	0.556
		MLMM	0.1	7.9	0.506
		FarmCPU	0.1	7.9	0.506
		CS-LMM	0.9	**7.4**	0.556
		WALD+FDR	**0.7**	1640.1	0.528
		Lasso	0.0	10.0	0.5
		AdaLasso	0.0	9.6	0.5
		PrecisionLasso	0.3	9.3	0.519
0.0001	25	LMM+FDR	0.4	7.8	0.525
		sparse LMM	0.5	7.5	0.531
		MLMM	0.1	7.9	0.506
		FarmCPU	0.1	7.9	0.506
		CS-LMM	**0.7**	**7.2**	**0.544**
		WALD+FDR	**0.8**	1860.6	0.532
		Lasso	0.0	10.0	0.5
		AdaLasso	0.0	9.8	0.5
		PrecisionLasso	0.1	9.3	0.506
0.001	25	LMM+FDR	0.5	14.9	0.531
		sparse LMM	0.6	7.3	0.537
		MLMM	0.1	7.9	0.506
		FarmCPU	0.1	7.9	0.506
		CS-LMM	**0.8**	**7.1**	**0.55**
		WALD+FDR	**1.2**	2611.7	**0.55**
		Lasso	0.0	10.2	0.5
		AdaLasso	0.0	9.9	0.5
		PrecisionLasso	0.0	9.8	0.5
0.005	25	LMM+FDR	0.5	43.0	0.531
		sparse LMM	0.6	7.7	0.537
		MLMM	0.1	7.9	0.506
		FarmCPU	0.1	7.9	0.506
		CS-LMM	0.8	**7.6**	0.55
		WALD+FDR	**0.4**	1963.5	0.506
		Lasso	0.0	10.1	0.5
		AdaLasso	0.0	9.4	0.5
		PrecisionLasso	0.1	8.9	0.506
0.0001	10	LMM+FDR	0.2	7.7	0.512
		sparse LMM	0.2	8.9	0.512
		MLMM	0.1	7.9	0.506
		FarmCPU	0.1	7.9	0.506
		CS-LMM	**0.4**	**7.2**	**0.525**
		WALD+FDR	**0.4**	2171.1	0.504
		Lasso	0.0	10.2	0.5
		AdaLasso	0.0	9.5	0.5
		PrecisionLasso	0.0	9.2	0.5
0.001	10	LMM+FDR	0.2	14.1	0.512
		sparse LMM	0.2	8.0	0.512
		MLMM	0.1	7.9	0.506
		FarmCPU	0.1	7.9	0.506
		CS-LMM	**0.4**	**7.7**	**0.525**
		WALD+FDR	**0.7**	3126.9	0.513
		Lasso	0.0	10.1	0.5
		AdaLasso	0.0	9.6	0.5
		PrecisionLasso	0.0	9.8	0.5
0.005	10	LMM+FDR	0.2	39.9	0.512
		sparse LMM	0.2	8.2	0.512
		MLMM	0.1	**7.9**	0.506
		FarmCPU	0.1	**7.9**	0.506
		CS-LMM	0.3	8.2	**0.519**

Table S10: The scenario of common variants with strong effects.

MAF	coefficient	model	TP	FP	auROC
0.01	50	WALD+FDR	**2.3**	2860.5	**0.62**
		Lasso	0.0	9.8	0.5
		AdaLasso	0.0	9.8	0.5
		PrecisionLasso	0.0	9.8	0.5
		LMM+FDR	1.5	48.6	0.593
		sparse LMM	1.0	7.8	0.562
		MLMM	0.1	7.9	0.506
		FarmCPU	0.1	7.9	0.506
		CS-LMM	1.1	**7.2**	0.569
0.05	50	WALD+FDR	**3.5**	6482.5	**0.671**
		Lasso	0.0	9.9	0.5
		AdaLasso	0.0	9.8	0.5
		PrecisionLasso	0.0	10.0	0.5
		LMM+FDR	2.4	73.8	0.649
		sparse LMM	1.4	7.5	0.587
		MLMM	0.1	7.9	0.506
		FarmCPU	0.1	7.9	0.506
		CS-LMM	1.7	**6.6**	0.606
0.1	50	WALD+FDR	**4.4**	8376.5	**0.723**
		Lasso	0.0	10.1	0.5
		AdaLasso	0.0	9.9	0.5
		PrecisionLasso	0.0	10.2	0.5
		LMM+FDR	3.3	39.5	0.706
		sparse LMM	2.1	6.9	0.631
		MLMM	0.1	7.9	0.506
		FarmCPU	0.1	7.9	0.506
		CS-LMM	2.0	**5.6**	0.625
0.01	25	WALD+FDR	**1.7**	3525.1	**0.573**
		Lasso	0.0	10.1	0.5
		AdaLasso	0.0	9.7	0.5
		PrecisionLasso	0.0	10.2	0.5
		LMM+FDR	0.7	56.8	0.543
		sparse LMM	0.6	7.4	0.537
		MLMM	0.1	7.9	0.506
		FarmCPU	0.1	7.9	0.506
		CS-LMM	0.6	**7.3**	0.537
0.05	25	WALD+FDR	**3.0**	7207.3	**0.63**
		Lasso	0.0	10.7	0.5
		AdaLasso	0.0	10.3	0.5
		PrecisionLasso	0.0	9.8	0.5
		LMM+FDR	1.5	61.5	0.593
		sparse LMM	0.9	7.8	0.556
		MLMM	0.1	7.9	0.506
		FarmCPU	0.1	7.9	0.506
		CS-LMM	0.9	**7.1**	0.556
0.1	25	WALD+FDR	**3.7**	10799.2	**0.652**
		Lasso	0.0	9.7	0.5
		AdaLasso	0.0	9.8	0.5
		PrecisionLasso	0.0	10.0	0.5
		LMM+FDR	1.8	41.0	0.612
		sparse LMM	1.2	6.8	0.575
		MLMM	0.1	7.9	0.506
		FarmCPU	0.1	7.9	0.506
		CS-LMM	1.7	**5.9**	0.606
0.01	10	WALD+FDR	**0.9**	3966.7	0.517
		Lasso	0.0	10.5	0.5
		AdaLasso	0.0	9.9	0.5
		PrecisionLasso	0.0	10.1	0.5
		LMM+FDR	0.2	63.2	0.512
		sparse LMM	0.5	7.9	0.531
		MLMM	0.1	7.9	0.506
		FarmCPU	0.1	7.9	0.506
		CS-LMM	0.5	**7.8**	**0.531**
0.05	10	WALD+FDR	**2.7**	9774.0	**0.572**
		Lasso	0.0	10.0	0.5
		AdaLasso	0.0	9.8	0.5
		PrecisionLasso	0.0	9.7	0.5
		LMM+FDR	0.3	88.1	0.518
		sparse LMM	0.2	8.2	0.512
		MLMM	0.1	7.9	0.506
		FarmCPU	0.1	7.9	0.506
		CS-LMM	0.4	**7.5**	0.525
0.1	10	WALD+FDR	**3.3**	14073.8	**0.581**
		Lasso	0.0	9.7	0.5
		AdaLasso	0.0	10.0	0.5
		PrecisionLasso	0.0	9.5	0.5
		LMM+FDR	0.5	46.7	0.531
		sparse LMM	0.4	8.0	0.525
		MLMM	0.1	7.9	0.506
		FarmCPU	0.1	7.9	0.506
		CS-LMM	0.7	**7.6**	0.544

Table S11: The scenario of rare variants with small effects.

MAF	coefficient	model	TP	FP	auROC
		WALD+FDR	0.2	1898.3	0.494
		Lasso	0.0	10.0	0.5
		AdaLasso	0.0	9.5	0.5
		PrecisionLasso	0.0	9.1	0.5
0.0001	5	LMM+FDR	0.1	7.8	0.506
		sparse LMM	0.2	8.6	0.512
		MLMM	0.1	7.9	0.506
		FarmCPU	0.1	7.9	0.506
		CS-LMM	**0.3**	**7.5**	**0.519**
		WALD+FDR	0.2	2112.8	0.492
		Lasso	0.0	10.0	0.5
		AdaLasso	0.0	9.5	0.5
		PrecisionLasso	0.0	9.4	0.5
0.001	5	LMM+FDR	0.1	14.3	0.506
		sparse LMM	0.2	8.2	0.512
		MLMM	0.1	7.9	0.506
		FarmCPU	0.1	7.9	0.506
		CS-LMM	**0.3**	**7.7**	**0.519**
		WALD+FDR	**0.3**	3086.5	0.488
		Lasso	0.0	10.0	0.5
		AdaLasso	0.0	9.5	0.5
		PrecisionLasso	0.0	9.8	0.5
0.005	5	LMM+FDR	0.1	39.5	0.506
		sparse LMM	0.2	8.3	0.512
		MLMM	0.1	7.9	0.506
		FarmCPU	0.1	7.9	0.506
		CS-LMM	**0.3**	**7.7**	**0.519**
		WALD+FDR	**0.1**	1989.9	0.486
		Lasso	0.0	10.0	0.5
		AdaLasso	0.0	9.5	0.5
		PrecisionLasso	0.0	9.1	0.5
0.0001	1.1	LMM+FDR	0.0	**7.8**	0.5
		sparse LMM	0.0	8.6	0.5
		MLMM	**0.1**	7.9	0.506
		FarmCPU	**0.1**	7.9	**0.506**
		CS-LMM	0.0	**7.8**	0.5
		WALD+FDR	**0.1**	2212.8	0.484
		Lasso	0.0	10.0	0.5
		AdaLasso	0.0	9.5	0.5
		PrecisionLasso	0.0	9.1	0.5
0.001	1.1	LMM+FDR	0.0	14.1	0.5
		sparse LMM	0.0	8.6	0.5
		MLMM	**0.1**	**7.9**	0.506
		FarmCPU	**0.1**	**7.9**	**0.506**
		CS-LMM	0.0	8.0	0.5
		WALD+FDR	**0.2**	3155.4	0.481
		Lasso	0.0	10.0	0.5
		AdaLasso	0.0	9.9	0.5
		PrecisionLasso	0.0	10.0	0.5
0.005	1.1	LMM+FDR	0.0	38.7	0.5
		sparse LMM	0.0	8.3	0.5
		MLMM	0.1	**7.9**	0.506
		FarmCPU	0.1	**7.9**	**0.506**
		CS-LMM	0.0	8.3	0.5

Table S12: The scenario of common variants with small effects.

MAF	coefficient	model	TP	FP	auROC
		WALD+FDR	**0.4**	4261.2	0.483
		Lasso	0.0	10.1	0.5
		AdaLasso	0.0	9.6	0.5
		PrecisionLasso	0.0	10.0	0.5
0.01	5	LMM+FDR	0.1	61.7	0.506
		sparse LMM	0.2	**7.7**	0.512
		MLMM	0.1	7.9	0.506
		FarmCPU	0.1	7.9	0.506
		CS-LMM	0.3	7.9	**0.519**
		WALD+FDR	**2.0**	10284.0	0.515
		Lasso	0.0	10.1	0.5
		AdaLasso	0.0	10.1	0.5
		PrecisionLasso	0.0	10.0	0.5
0.05	5	LMM+FDR	0.1	105.8	0.505
		sparse LMM	0.2	**7.6**	0.512
		MLMM	0.1	7.9	0.506
		FarmCPU	0.1	7.9	0.506
		CS-LMM	0.3	7.8	**0.519**
		WALD+FDR	**2.5**	13270.8	**0.535**
		Lasso	0.0	9.8	0.5
		AdaLasso	0.0	9.9	0.5
		PrecisionLasso	0.0	9.6	0.5
0.1	5	LMM+FDR	0.1	62.8	0.506
		sparse LMM	0.1	8.4	0.506
		MLMM	0.1	7.9	0.506
		FarmCPU	0.1	7.9	0.506
		CS-LMM	0.5	**7.8**	0.531
		WALD+FDR	**0.4**	4270.3	0.482
		Lasso	0.0	10.0	0.5
		AdaLasso	0.0	9.8	0.5
		PrecisionLasso	0.0	9.8	0.5
0.01	1.1	LMM+FDR	0.0	62.8	0.499
		sparse LMM	0.0	8.4	0.5
		MLMM	0.1	**7.9**	0.506
		FarmCPU	0.1	**7.9**	**0.506**
		CS-LMM	0.0	8.3	0.5
		WALD+FDR	**1.5**	10131.7	0.488
		Lasso	0.0	9.9	0.5
		AdaLasso	0.0	10.1	0.5
		PrecisionLasso	0.0	10.5	0.5
0.05	1.1	LMM+FDR	0.0	110.4	0.499
		sparse LMM	0.0	8.5	0.5
		MLMM	0.1	**7.9**	**0.506**
		FarmCPU	0.1	**7.9**	0.506
		CS-LMM	0.0	8.4	0.5
		WALD+FDR	**2.5**	13038.2	**0.518**
		Lasso	0.0	9.7	0.5
		AdaLasso	0.0	10.0	0.5
		PrecisionLasso	0.0	9.7	0.5
0.1	1.1	LMM+FDR	0.0	72.8	0.499
		sparse LMM	0.0	8.4	0.5
		MLMM	0.1	**7.9**	0.506
		FarmCPU	0.1	**7.9**	0.506
		CS-LMM	0.0	8.2	0.5

References
